# Constitutive Model Parameter Identification Based on Optimization Method and Formability Analysis for Ti6Al4V Alloy

**DOI:** 10.3390/ma15051748

**Published:** 2022-02-25

**Authors:** Xuewen Chen, Bo Zhang, Yuqing Du, Mengxiang Liu, Rongren Bai, Yahui Si, Bingqi Liu, Dong-Won Jung, Akiyoshi Osaka

**Affiliations:** 1 School of Materials Science and Engineering, Henan University of Science and Technology, 263 Kaiyuan Avenue, Luoyang 471023, China; zhangbo32103@126.com (B.Z.); duyqstephanie@163.com (Y.D.); luimx96@163.com (M.L.); bairongren317@163.com (R.B.); siyahui@stu.haust.edu.cn (Y.S.); lbq8565@163.com (B.L.); akiyoshiosaka@icloud.com (A.O.); 2 Faculty of Mechanical and System Engineering, Jeju National University, Jeju-si 63243, Korea; 3 Institute of Engineering, Okayama University, Tsushima, Okayama 700-8530, Japan

**Keywords:** Ti6Al4V alloy, response surface method, reverse optimization, high-temperature constitutive model, formability, hot processing map

## Abstract

Titanium alloy is widely applied in aerospace, medical, shipping and other fields due to its high specific strength and low density. The purpose of this study was to analyze the formability of Ti6Al4V alloys at elevated temperatures. An accurate constitutive model is the basic condition for accurately simulating the plastic forming of materials, and it is an important basis for optimizing the parameters of the hot forging forming process. In this study, the optimization algorithm was used to accurately identify the high-temperature constitutive model parameters of Ti6Al4V titanium alloy, and the hot working diagram was established to optimize the hot forming process parameters. The optimal forming conditions of Ti6Al4V titanium alloy are given. Ti6Al4V alloy was subjected to high-temperature compression tests at 800–1000 °C and at strain rates of 0.01–5 s^−1^ on a Gleeble-1500D thermal/mechanical simulation machine. Each parameter of the Hansel–Spittel constitutive model was taken as an independent variable, and the accumulated error between the stress calculated by the constitutive model and the stress obtained by experimentation was used as an objective function. Based on response surface methodology, an inverse optimization method for identifying the parameters of the high-temperature constitutive model of Ti6Al4V alloy is proposed in this paper. An orthogonal test design was adopted to obtain sample point data, and a third-order response surface approximate model was established. The genetic algorithm (GA) was applied to reversely optimize the parameters of the constitutive model. To verify the accuracy of the optimized constitutive model, the average absolute relative error (AARE) and correlation coefficient (R) were used to evaluate the reliability of optimized constitutive model. The R value of the model was 0.999, and the AARE value was 0.048, respectively, indicating that the established high-temperature constitutive model for Ti6Al4V alloy has good calculation accuracy. The flow stress behavior of the material could be accurately delineated. Meanwhile, in order to study the formability of Ti6Al4V alloy, the hot processing map of the alloy, based on a dynamic material model, was established in this paper. The optimum hot working domains of the Ti6Al4V alloy were determined within 840–920 °C/0.01–0.049 s^−1^ and 940–980 °C/0.11–1.65 s^−1^; the hot processing map was verified in combination with the microstructure, and the fine and equiaxed grains and a large amount of β phase could be found at 850 °C/0.01 s^−1^.

## 1. Introduction

As a representative α + β two-phase titanium alloy, Ti6Al4V has been widely applied in aerospace, biomedicine and other fields because of its characteristics of moderate density, high strength and good fracture toughness [1], and has been used to produce aerospace compressor blades [2], undercarriage components [3] and artificial bones [4]. However, the crystal structure of Ti6Al4V alloy at room temperature is a hexagonal close-packed structure (HCP), its plasticity and formability are poor, and its deformation resistance is large [5]. Therefore, it is not suitable for plastic processing [6] at room temperature. However, the formability of Ti6Al4V alloy at elevated temperatures can be improved. The constitutive model of materials is an expression used to describe the relationship between stress and various deformation conditions under operating conditions [7]. It can be used for the finite element simulation and optimization of deformation process, and the prediction of material flow behavior under different forming conditions, which is of great significance in terms of improving product quality and reducing costs. The inaccurate identification of constitutive model parameters will seriously affect the accuracy of finite element simulation results obtained for titanium alloy-based hot forming. Therefore, an accurate constitutive model needs be established to more deeply study the formability of Ti6Al4V alloy and to design a relevant thermal forging technology.

To predict the flow behavior of materials [8], constitutive models have been established; these mainly include the physics-based constitutive model, the phenomenological constitutive model, etc. The physics-based constitutive model focuses on describing the microscopic mechanism of the change process and can well explain the root cause of changes in flow stress [9]. Therefore, many physics-based models have been improved to accurately delineate the flow behavior of materials, such as the Mechanic Threshold Stress model [10], the Bammann-Chiesa-Johnson model [11], etc. However, the complexity of these models limits their application [12]. Empirical constitutive models do not need to consider all the physical phenomena during the deformation, and thus, it is intuitive and easy to define parameters using these models. Some are applied to simulate the plastic deformation process of alloys, such as the Arrhenius, Johnson-Cook and Hansel-Spittel models. The Arrhenius constitutive model was proposed by Sellars [13] to delineate the thermal deformation of metals and has been widely used. Ahmed Mosleh [14] used the Arrhenius model to describe the flow stress of near-α titanium alloy, and the correlation coefficient reached 0.9525, which verified the effectiveness of the Arrhenius model. Johnson and Cook [15] proposed the Johnson-Cook model, which is used to construct flow stress models under high-temperature, large-strain and high-strain-rate conditions. This model is not suitable for deformation under small strain and low strain rates. By considering the coupling effect of work hardening and thermal softening, Ravindranadh Bobbili et al. [16] improved the Johnson-Cook model to delineate the flow stress of the dynamic compression of Ti-13Nb-13Zr. The results indicate that the improved Johnson-Cook model has good predictive ability. In order to compare the predictive precision of the strain-compensated Arrhenius and improved Johnson–Cook model, H. R. Rezaei Ashtiani [17] adopted these two constitutive models to delineate the hot flow behavior of AA7022-T6 alloy under various plastic conditions; the linear correlation coefficients of the two models were 0.9914 and 0.9972, respectively, indicating that the Arrhenius model has better predictive ability. When considering the impact of strain on stress, reaching solutions using constitutive models requires a lot of work, and the prediction error is also large. To accurately describe the elevated temperature constitutive relationship of Mg-9Li-3Al-2Sr-2Y alloy, Guobing Wei [18] used the strain-compensated Arrhenius model, the Hansel-Spittel model and other constitutive models to make predictions, and the evaluation found that Hansel-Spittel model had the best predictive accuracy. This model takes into account the combined effects of strain rates and temperatures, which is applicable to a wider range. Therefore, the Hansel-Spittel model was used to delineate the thermal deformation behavior of Ti6Al4V alloy at high temperature.

Whether the parameters of constitutive models can be accurately identified is the main factor that affects the accuracy of finite element calculation. At present, traditional calibration methods are mostly used to solve the parameters of the Hansel-Spittel model. The calculated parameters, such as the average method, will lead to large errors in the calculated constitutive model. In recent years, optimization methods have been widely used in parameter identification. Iterating according to the objective function can identify parameters more accurately than the traditional average method. Kyunghoon Lee [19] determined nine parameters of the Hansel-Spittel model through the deterministic least-square method and the statistical maximum-likelihood method. The results showed that the traditional method for determining the parameters of the Hanse-Spittel model ignores the deterministic trend of the residual error, and the error is large. There is room for further improvement in terms of accuracy. Zhenglong Liang [20] assumed that the strain index in the Hansel-Spittel model was a polynomial function of the strain rate, combined its influence into the Hansel-Spittel model, and obtained parameters by means of traditional nonlinear analysis, thus improving the predictive precision of the Hansel-Spittel model. However, the traditional mathematical method is not only complicated, but it is also difficult to calculate its parameters, which will reduce the precision of the constitutive model. Thus, it is necessary to propose a new method that can accurately define the parameters of the Hansel-Spittel model. At present, few scholars have applied optimization techniques to the establishment of the parameters of the Hansel-Spittel model.

Reverse optimization is a solving process in which an error function is obtained by comparing experimental data of hot compression with calculated data, and the value of the error function is minimized [21]. The parameters are determined using an inverse optimization algorithm, and the obtained constitutive model has high precision. Optimization techniques can significantly improve the predictive accuracy of the material constitutive model. Michael Oluwatosin Bodunrin [22] found that the constitutive models of titanium alloy obtained by traditional methods had high error rates. The constitutive model parameters were optimized using a generalized reduction gradient method. The average absolute relative error was reduced by 30–40%. This shows that the optimization method has great advantages in terms of parameter identification.

As a common optimization method, the response surface method (RSM) uses experimental design to obtain necessary data, and then adopts a multiple-regression equation to fit the relationship between factors and response values; it is suitable for solving complex nonlinear problems. Response surface methodology was initially proposed by Box [23]; after that, it was constantly enriched and modified for different purposes. M. Abbasi [24] used the center-grouping method (CCD) combined with response surface modeling to invert Gurson-Tvergaard-Needleman (GTN) plastic-damage model parameters, and this achieved good results. Andrej Skrlec et al. [25] used two experimental design methods (the full factor method and the Taguchi orthogonal table) and the response surface method to identify Cowper–Symonds material model parameters for E185 steel. By comparison, it was found that the Taguchi orthogonal design approach could find the same or a similar optimal neighborhood in a shorter calculation time. It is suitable for identifying a large number of parameters and their domain range is very wide. Sun et al. [26] proposed an inversion technique to determine the material parameters of TB5 alloy using quasi-Newton and response surface methods, and found that the anisotropic parameters obtained by inversion could better describe the mechanical behavior of the material. Previous studies showed that the RSM method has the potential for successful application in parameter identification. Therefore, the RSM method was used to inversely identify the parameters of the high-temperature Hansel-Spittel model of Ti6Al4V.

The hot processing map can be used to further evaluate the machining properties of materials based on the flow stress [27]. It can reflect the processing properties of materials under different deformation conditions, predict the deformation of materials under various thermal processing conditions and analyze their microstructural characteristics. Prasad et al. established a dynamic material model (DMM) [28] to describe the plastic forming of materials during thermal deformation. At present, DMM is used to construct processing maps of different materials, including aluminum alloy, titanium, magnesium, aluminum, Ni-based alloy, steel, etc., optimizing the processing route [29]. The processing map can show the microstructural mechanisms of metal materials, avoid unstable domains, ensure the stability of materials, and select reasonable processing parameters [30]. Madan Patnamsetty [31] established the hot processing map of CoCrFeMnNi high-entropy alloy using the DMM, and obtained the optimal processing parameters of the alloy as follows: temperature 950–1100 °C, strain rate 0.01–0.5 s^−1^. H. Khorshidi et al. [32] developed processing map of stainless steel, and found that a fully refined dynamic recrystallization structure could be obtained at 1000–1200 °C and strain rates of 0.01–1 s^−1^. P. L. Narayana [33] constructed a processing map of Ti-19Al-22Mo and found that the processing performance was the best at 1100 °C and 0.01 s^−1^. Wedge cracking and localized material flow were the main instability characteristics in the process of thermal deformation. Therefore, the DMM based hot processing map can be used to further determine the optimal process parameters effectively.

Ti6Al4V alloy has poor plasticity at room temperature, and its formability can be improved by increasing the temperature. The constitutive model is an important basis and premise for analyzing its formability. Therefore, in order to accurately identify the constitutive model parameters describing the high-temperature flow stress behavior of Ti6Al4V, a hot compression test of Ti6Al4V alloy was carried out in this paper, and a parameter definition method based on the inverse optimization algorithm of the constitutive model was proposed. The precision of the constitutive model was further evaluated by comparing the calculated stress with the experimental stress using standard statistical parameters. To optimize the process parameters, the hot processing map of Ti6Al4V was established on the basis of the dynamic material model, and the optimal processing conditions were determined.

## 2. Materials and Methods

**Figure 1:**
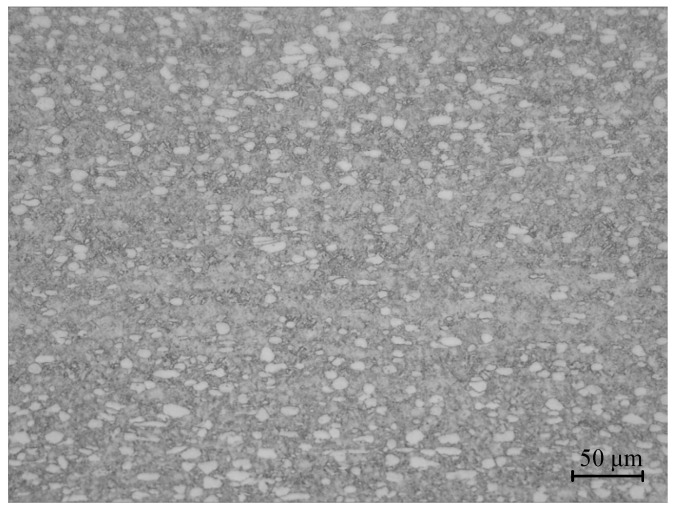
Original microstructure of Ti6Al4V.

**Figure 2:**
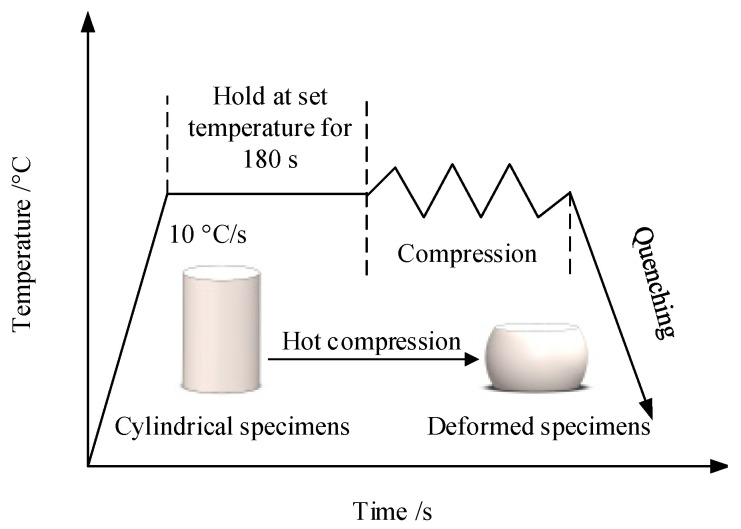
Test process scheme of Ti6Al4V.

In this study, the experimental material was forged Ti6Al4V alloy. The original microstructure, under optical microscopy, is shown in Figure 1; it consisted of an equiaxed α phase, an elongated α phase and an intergranular β phase. The chemical composition of Ti6Al4V alloy was determined by spectral analysis, and Table 1 illustrates the chemical composition of Ti6Al4V. The samples were processed into ø 8 mm × 12 mm cylinders, and thermal compression tests were performed using a Gleeble-1500D simulation machine (Dynamic Systems Inc, Poughkeepsie, NY, USA) in vacuum conditions. Before compression, graphite was evenly daubed on the two ends of the specimen that were in contact with the pressure head of the simulator to play the role of a lubricant, so as to reduce or eliminate the influence of friction on the hot compression test, and then make the specimen deformation as uniform as possible. The deformation amount of the compressed sample was 50%. The deformation temperatures were 800 °C, 850 °C, 900 °C, 950 °C and 1000 °C, and the strain rates were 0.01 s^−1^, 0.1 s^−1^, 1 s^−1^ and 5 s^−1^. The temperatures of samples were measured by platinum rhodium thermocouples. These samples were heated to specified compression temperatures of 10 °C/s, then kept for 3mins to eliminate the temperature gradients inside the samples. Finally, the compression test was carried out at the predetermined strain rate. To preserve the microstructure, the samples were immediately quenched in water when deformation reached 50%. Finally, an optical microscope (Olympus-pmg3) (OlympusCorporation, Tokyo, Japan) was used to observe the metallographic structure. The test scheme is shown in Figure 2.

## 3. Results and Discussion

### 3.1. Flow Stress Curves for Ti6Al4V in High-Temperature Compression Test

**Figure 3:**
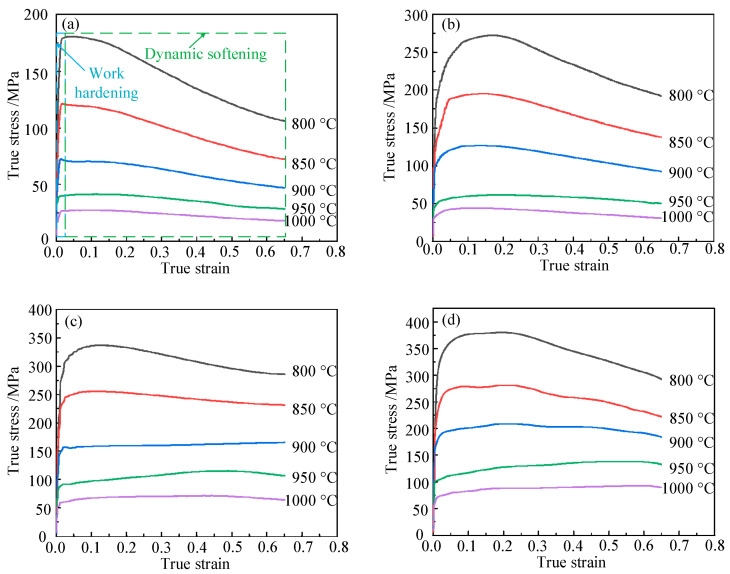
True stress–strain curves of Ti6Al4V: (**a**) $\dot{\varepsilon }$ = 0.01 s^−1^; (**b**) $\dot{\varepsilon }$ = 0.1 s^−1^; (**c**) $\dot{\varepsilon }$ = 1 s^−1^; (**d**) $\dot{\varepsilon }$ = 5 s^−1^.

Typical flow stress curves of Ti6Al4V under various thermomechanical conditions are shown in Figure 3. According to Figure 3, the change of stress with strain went through two stages: work hardening (blue box part in Figure 3) and softening (green box part in Figure 3).

As shown in Figure 3a, the flow stress curve at 800 °C/0.01 s^−1^ was a typical dynamic recrystallization curve. At the micro-strain stage from 0 to 0.035, the stress increased sharply, reaching 180.634 MPa. This was mainly due to accumulation of work hardening and deformation energy. After the stress increased to its peak value, it gradually reduced, and the value remained at about 106.022 MPa. This was because at 800 °C, the Ti6Al4V alloy was dominated by α phase, and the α-phase grains had a hexagonal close-packed structure. This structure had a low stacking fault energy and was prone to dynamic recrystallization; furthermore, there were fewer slip systems, and a large amount of deformation energy was produced during deformation. The deformation energy was transformed into distortion energy, then dynamic recrystallization occurred, dynamic softening was greater than strain hardening, and the curve showed a downward trend.

As presented in Figure 3c, when the temperature was 900 °C and strain rate was 1 s^−1^, and the flow stress curve was affected by dynamic recovery. This was because the deformation of the material at a high strain rate (1 s^−1^) was faster and the recrystallization time was shorter. Moreover, a large amount of the α phase (densely packed hexagonal structure) in the microstructure had been transformed into β phase (body-centered cubic structure) at 900 °C. Dynamic recovery characteristics were observed under the influence of high strain rate and β phase. The flow stress curve was a dynamic recovery curve when the temperature was 1000 °C and the strain rate was 1 s^−1^. The stress first increased rapidly to 58.331 MPa, then slowly increased to 69.724 MPa with the increasing of the strain. This was because at 1000 °C, the internal structure of the Ti6Al4V alloy was dominated by β phase (body-centered cubic structure), with high stacking fault energy and more slip systems, and the deformation energy was not enough to produce dynamic recrystallization. When the temperature was 1000 °C and the strain rate was increased to 5 s^−1^, as shown in Figure 3d, the deformation speed was faster, the dynamic recrystallization time was shorter, and the dynamic recrystallization was less obvious, so the flow stress curve also showed dynamic recovery characteristics.

**Figure 4:**
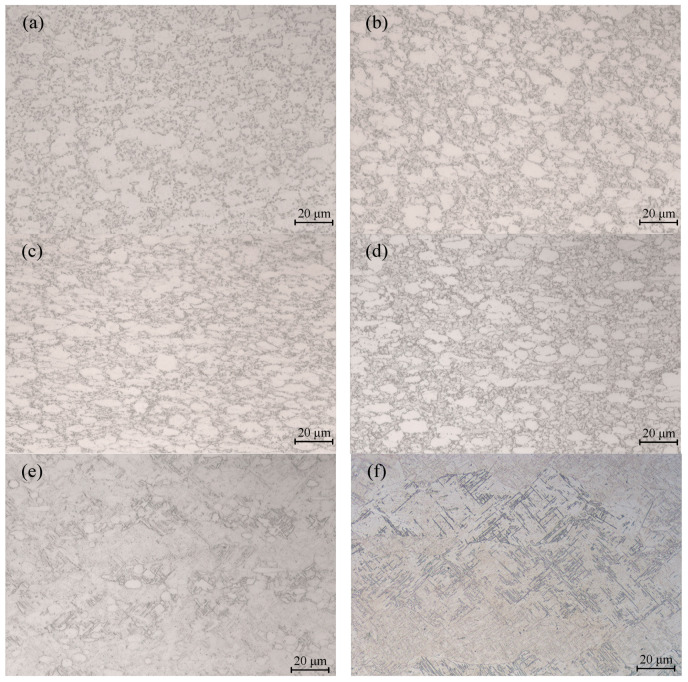
Microstructure of Ti6Al4V under various deformation conditions: (**a**) 800 °C/0.01 s^−1^; (**b**) 850 °C/0.01 s^−1^; (**c**) 850 °C/5 s^−1^; (**d**) 900 °C/0.01 s^−1^; (**e**) 950 °C/0.01 s^−1^; (**f**) 1000 °C/0.01 s^−1^.

The microstructure of Ti6Al4V was observed by optical microscope. As can be seen in Figure 4, the flow stress behavior after dynamic softening was evident in the metallographic structure of the Ti6Al4V alloy. Figure 4a shows the metallographic structure at 800 °C/0.01 s^−1^. The microstructure contained relatively fine α phase equiaxed grains due to dynamic recrystallization. When the temperature increased to 850 °C, as illustrated in Figure 4b, dynamic recrystallization was more complete and the α-phase grain size was larger. When the Ti6Al4V alloy was compressed at 850 °C/5 s^−1^, more α-phase grains in the microstructure were flattened and elongated into fine strips, but there were also smaller equiaxed α-phase grains, and the sizes of the equiaxed grains were smaller than the grains observed at a strain rate of 0.01 s^−1^. This was because the deformation time of materials is shorter at high strain rates. It was found that the dynamic recrystallization was insufficient. Therefore, an appropriate strain rate and temperature are favorable for the dynamic recrystallization of Ti6Al4V alloy.

When the temperature increased from 850 °C to 900 °C and the strain rate was 0.01 s^−1^, the content of the β phase in the Ti6Al4V alloy clearly increased. At 950 °C/0.01 s^−1^, there was more α-phase transformed into β-phase. There were few α-phase equiaxed grains and a lot of β-phase tissues in the tissue. When the temperature increased to 1000 °C, the α phase was completely transformed into β phase, and then the coarse β grains were formed because the deformation energy was not enough for dynamic recrystallization to occur.

### 3.2. Parameter Identification Method of Elevated Temperature Constitutive Model for Ti6Al4V

#### 3.2.1. Orthogonal Experimental Design of Hansel–Spittel Constitutive Model for Ti6Al4V

To research the flow characteristics of Ti6Al4V, it was necessary to conduct a constitutive analysis. The constitutive model can quantitatively describe the quantitative relationship among the stress, strain, strain rate and temperature. The Hansel-Spittel model considers the impact of the strain, strain rate and temperature on the stress, which has a wide range of applicability and can reflect the thermal deformation behavior of Ti6Al4V more accurately. The Hansel-Spittel model [34,35,36] is shown in Equation (1):

(1) $$\sigma =A\cdot e^{m_{1}T}\cdot \varepsilon^{m_{2}}\cdot {\dot{\varepsilon }}^{m_{3}}\cdot e^{\frac{m_{4}}{\varepsilon }}\cdot {\left(1+\varepsilon \right)}^{m_{5}T}\cdot e^{m_{7}\varepsilon }\cdot {\dot{\varepsilon }}^{m_{8}T}\cdot T^{m_{9}}$$

where A is the material constant; m_1_ is the temperature correlation coefficient; m_2_ is the strain-strengthening index; m_3_ is the strain-rate-strengthening index; m_4_ is the strain-softening coefficient; m_5_ represents the coefficient of temperature and strain coupling; m_7_ represents the strain-strengthening coefficient; m_8_ represents the coefficient of strain rate and temperature coupling; m_9_ represents the temperature-strengthening index.

Comparing the experimental stress with the calculated stress, their similarity was evaluated. The accumulated error between the stress calculated by the constitutive model and the stress obtained experimentally was taken as the objective function, as shown in Equation (2):

(2) $$O(f)=\frac{\sum_{i}^{n}{\left(\sigma_{i}^{\exp}-\sigma_{i}^{\mathrm{cal}}\right)}^{2}}{\sum_{i}^{n}{\left(\sigma_{i}^{\exp}\right)}^{2}}$$

where $\sigma_{i}^{\exp}$ represents the stress of the *i*th data in the experiment, and $\sigma_{i}^{\mathrm{cal}}$ represents the stress of the *i*th data in the calculation. The closer O(f) is to 0, the closer the calculated results are to the experimental results.

**Figure 5:**
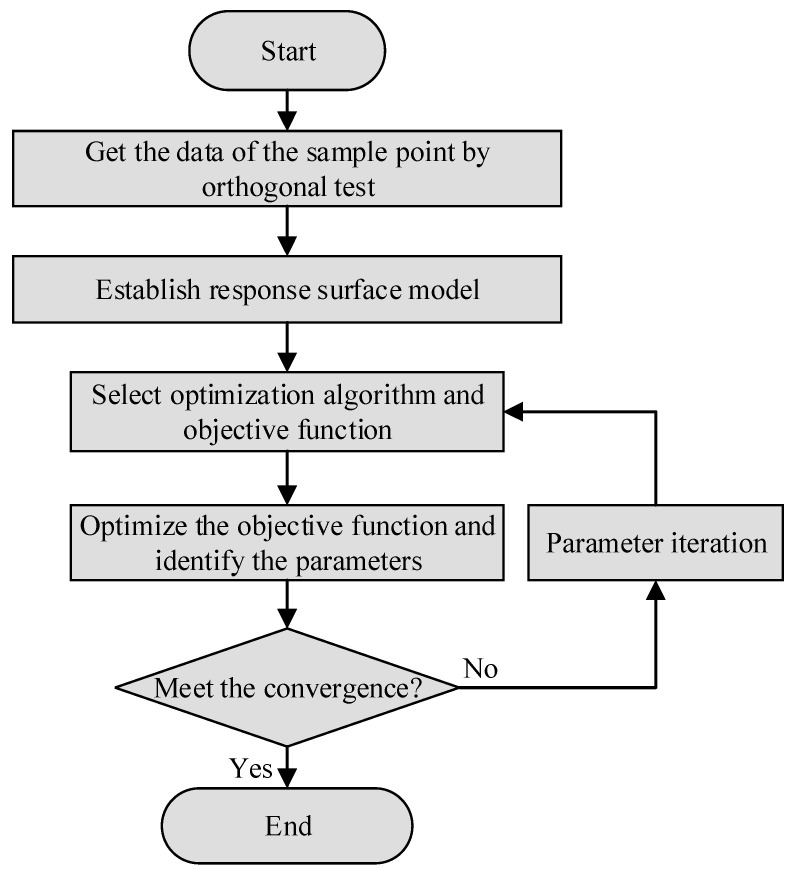
Parameter identification flow chart.

To solve the problem of the difficulty in solving the parameters of the Hansel-Spittel model, this paper proposes a new method to identify the parameters of the Hansel-Spittel model of Ti6Al4V using response surface technology. The parameter-identification process is shown in Figure 5:

Using an orthogonal experimental design, and standardization of the orthogonal table to arrange the testing scheme, the sample points were distributed in the design space according to their orthogonality; these points had the characteristics of “uniform dispersivity, symmetrical comparability” relative to the research target in terms of the response relationship between input and output. Therefore, the orthogonal experiment is a kind of high efficiency, rapid test-design method [37].

The uncertainty of the experimental model was due to the lack of experimental information caused by the small number of orthogonal experimental groups. In order to reduce the uncertainty of the experimental model, enough orthogonal experiments needed to be carried out.

The Hansel-Spittel constitutive model has nine parameters; to obtain sufficient data to establish the response surface model [38], the L_32_ (4^9^) orthogonal table was selected for the experimental design in this study, and the feasible region was defined based on the parameters of the Hansel-Spittel model. Namely, the maximum and minimum values of the parameters of the Hansel-Spittel model were obtained by fitting each stress–strain curve to determine their variation range. The objective function was the cumulative error O(f) between the calculated stress and the experimental stress. The factor levels were determined as shown in Table 2.

L_32_ (4^9^) is an orthogonal table with nine factors and four levels. A total of 32 groups of experiments needed to be conducted, and each group of experiments was arranged according to the group number from 1 to 32. The parameter values of each group of tests were substituted into the Hansel-Spittel model to obtain the calculated stress values of this group, and then the value of the objective function was obtained using Equation (2). Table 3 shows the orthogonal test results.

#### 3.2.2. Establishment of Response Surface Model for Constitutive Relation of Ti6Al4V at High Temperature

RSM [39] can use appropriate iterative methods to fit sample points in the design space and gradually approximate them instead of real responses, presenting the complex relationship between research objects and design variables through mathematical expressions [40]. As an optimization method of mathematical statistics, the response of RSM depends on a number of important variables to model and optimize the response [41]. It expresses the relationships between responses and parameters by approximating the construction of a polynomial with explicit expression.

Multivariate nonlinear regression fitting was carried out on the orthogonal test data, and the established response surface model function was:

(3) $$\begin{array}{ll} O(f)= & -5431-2.29A-1.17\times 10^{6}m_{1}+2709m_{2}-3.6\times 10^{4}m_{3}+2.1\times 10^{6}m_{4}+\\ & 2.1\times 10^{5}m_{5}-6.2m_{7}+7.6\times 10^{5}m_{8}-147m_{9}+2960m_{2}^{2}+7.3\times 10^{4}m_{3}^{2}+\\ & 3.3\times 10^{8}m_{4}^{2}-5.6\times 10^{5}m_{5}^{2}-1.1m_{7}^{2}-1.9\times 10^{9}m_{8}^{2}+70m_{9}^{2}-213{\mathrm{Am}}_{1}-\\ & 2.3{\mathrm{Am}}_{2}+5{\mathrm{Am}}_{3}+286{\mathrm{Am}}_{4}+118{\mathrm{Am}}_{5}+2121{\mathrm{Am}}_{8}+1.6\times 10^{6}m_{1}m_{2}-\\ & 5.3\times 10^{6}m_{1}m_{3}+2.6\times 10^{7}m_{1}m_{5}-31510m_{2}^{3}+1.2\times 10^{5}m_{3}^{3} \end{array}$$

Table 4 presents the results of the variance analysis. DOF is degrees of freedom, MS represents the mean square, and SS is the sum of squares. It can be seen that the linear terms m_1_, m_2_, m_4_, m_5_ and m_9_ had significant responses to O(f), the square terms m_4_ and m_8_ had significant responses to O(f), the interaction terms Am_3_, Am_5_, m_1_m_2_, m_1_m_3_ and m_1_m_5_ had significant responses to O(f) and the cubic terms m_2_ and m_3_ had significant responses to O(f). The low *p* value of regression (*p* < 0.05) and large *R*^2^ (0.9952) indicated that the model fitted well and, thus, is suitable for identifying the parameters of the constitutive model.

**Figure 6:**
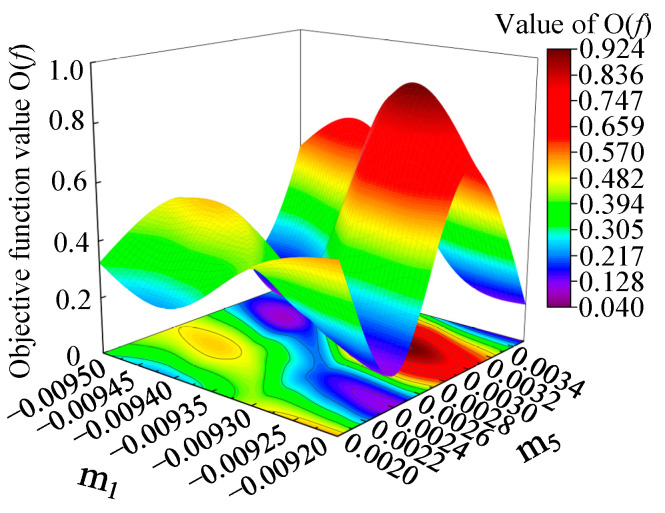
Response surface of the influence of parameters m_1_ and m_5_ on the objective function value O(f).

In this paper, m_1_m_5_ among the five significant interaction terms determined by variance analysis, was taken as an example to establish the response surface diagrams of m_1_ and m_5_, and to study their effects on the objective function O(f), as shown in Figure 6. According to the response surface, it can be seen that parameters m_1_ and m_5_ had a significant influence on the value of O(f). There were two minimum value regions in the response surface, which appeared in the lower m_1_ value (−0.0095 < m_1_ < −0.00935) and higher m_5_ value (0.0028 < m_5_ < 0.0032), and the higher m_1_ value (−0.0093 < m_1_ < −0.0092) and lower m_5_ value (0.0022 < m_5_ < 0.0025), respectively.

#### 3.2.3. Identification of High-Temperature Hansel-Spittel Model Parameters of Ti6Al4V by Response Surface Reverse Optimization Method

The genetic algorithm (GA) is an optimization algorithm proposed by Prof. J.H. Holland that simulates biological inheritance and follows the survival rules of nature [42]. It produces an optimal solution according to the principle of natural selection [37].

The functional relationship among the parameters and the objective function was established using the response surface method, and then GA was used to identify the parameters of the constitutive model [43]. Firstly, an initial value was assigned to each parameter. GA was selected as the optimization method to minimize the objective function O(f). O(f) could evaluate the error among the experimental stress and the calculated stress of the constitutive model. When O(f) was the smallest, the corresponding parameter was taken to be the optimal solution. The optimal parameters were obtained using the response surface method, as shown in Table 5.

By applying the parameters optimized above to Equation (1), the Hansel-Spittel model of Ti6Al4V alloy could be obtained, as shown in Equation (4):

(4) $$\sigma =356.307\cdot e^{-0.00935T}\cdot \varepsilon^{-0.032}\cdot {\dot{\varepsilon }}^{-0.2007}\cdot e^{\frac{-0.0033}{\varepsilon }}\cdot {\left(1+\varepsilon \right)}^{0.00317T}\cdot e^{-2.356\varepsilon }\cdot {\dot{\varepsilon }}^{0.00041T}\cdot T^{1.089}$$

#### 3.2.4. Verification of High-Temperature Hansel-Spittel Model of Ti6Al4V

**Figure 7:**
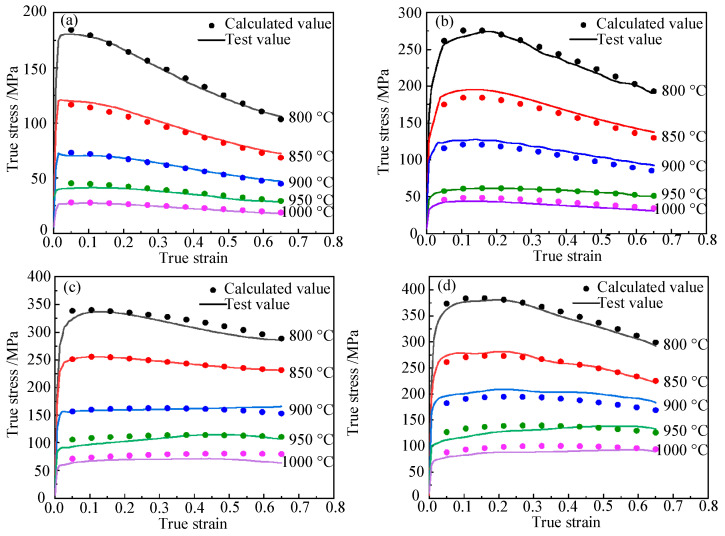
Comparison of calculated values of the improved Hansel–Spittel model and experimental values: (**a**) $\dot{\varepsilon }$ = 0.01 s^−1^; (**b**) $\dot{\varepsilon }$ = 0.1 s^−1^; (**c**) $\dot{\varepsilon }$ = 1 s^−1^; (**d**) $\dot{\varepsilon }$ = 5 s^−1^.

The flow stress was calculated under different conditions using the established high-temperature constitutive model. A comparison of the calculated and experimental flow curves at various temperatures is shown in Figure 7.

The reliability of the Hansel-Spittel model at high temperatures was verified by means of the average absolute relative error (AARE) and the correlation coefficient (R). They can be expressed as Equations (5) and (6):

(5) $$R=\frac{\sum_{i-1}^{N}\left(\sigma_{i}^{\exp}-\bar{\sigma^{\exp}}\right)\left(\sigma_{i}^{\exp}-\bar{\sigma^{\mathrm{cal}}}\right)}{\sqrt{{\sum_{i=1}^{N}\left(\sigma_{i}^{\exp}-\bar{\sigma^{\exp}}\right)}^{2}}\sqrt{{\sum_{i=1}^{N}\left(\sigma_{i}^{\exp}-\bar{\sigma^{\mathrm{cal}}}\right)}^{2}}}$$

(6) $$AARE\left(\%\right)=\frac{1}{N}\sum_{i=1}^{N}\left|\frac{\sigma_{i}^{\exp}-\sigma_{i}^{\mathrm{cal}}}{\sigma_{i}^{\exp}}\right|\times 100$$

In these equations, $\sigma_{i}^{\exp}$ represents the experimental stress, $\sigma_{i}^{\mathrm{cal}}$ represents the calculated stress; $\bar{\sigma^{\exp}}$ and $\bar{\sigma^{\mathrm{cal}}}$ represent the average values of $\sigma_{i}^{\exp}$ and $\sigma_{i}^{\mathrm{cal}}$, respectively. *N* represents the number of data.

**Figure 8:**
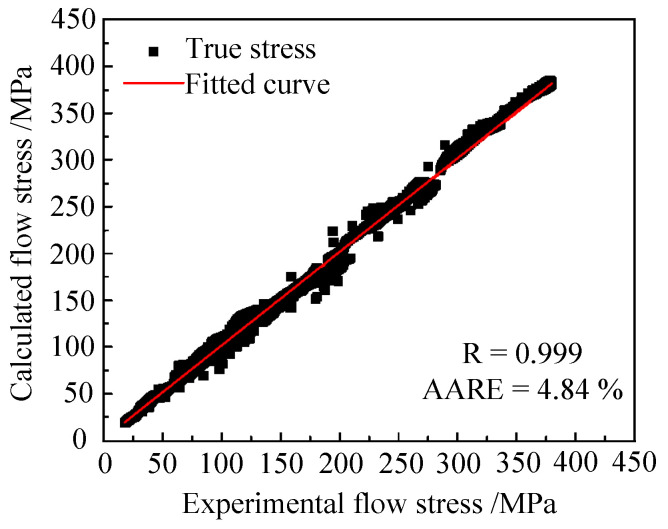
Relationship between experimental and calculated flow stress values.

The R and AARE values of the Hansel-Spittel model were 0.9999 and 0.0484, as shown in Figure 8. Therefore, the model can accurately delineate the flow behavior of Ti6Al4V at elevated temperatures.

### 3.3. Hot Processing Map of Ti6Al4V

In order to design an optimal plastic forming process for Ti6Al4V alloy, it was necessary to research forming conditions of its hot working. A processing map based on the dynamic material model (DMM) was superimposed on a power dissipation diagram and flow instability diagram, which made it easy to observe the distribution of the power dissipation coefficient under different deformation conditions and avoid flow instability caused by adiabatic shear bands [44].

Establishing the hot processing map according to DMM was an important step to optimize the thermal machining conditions and control the microstructure. According to the theory of DMM, the power loss of the workpiece during hot deformation is made up of the power dissipated by plastic processing, the power dissipated by superplastic flow, the power dissipated by dynamic recrystallization and other types of microstructural evolution, as follows [28]:

(7) $$P=\sigma \dot{\varepsilon }=G+J=\int_{0}^{\dot{\varepsilon }}\sigma d\dot{\varepsilon }+\int_{0}^{\sigma }\dot{\varepsilon }d\sigma$$

where, *P* is the power loss, σ is the flow stress, $\dot{\varepsilon }$ is the strain rate, *G* is the dissipated power for plastic deformation, *J* is the dissipated power by the microstructure evolution.

Under constant strain and temperature, the flow stress is Equation (8) as follows:

(8) $$\sigma =K{\dot{\varepsilon }}^{m}$$

*K* is a constant and *m* is the strain rate coefficient, which can be calculated using Equation (9):

(9) $$m=\frac{dJ}{dD}=\frac{\dot{\varepsilon }d\sigma }{\sigma d\dot{\varepsilon }}=\frac{d\ln\sigma }{l\ln\dot{\varepsilon }}$$

The power consumption efficiency (*η*) can be obtained using *m*:

(10) $$\eta =\frac{2m}{m+1}$$

In addition, the instability criterion for judging unstable regions can be obtained using Equation (11):

(11) $$\xi (\dot{\varepsilon })=\frac{\partial \log\frac{m}{m+1}}{\partial \log\dot{\varepsilon }}+m<0$$

**Figure 9:**
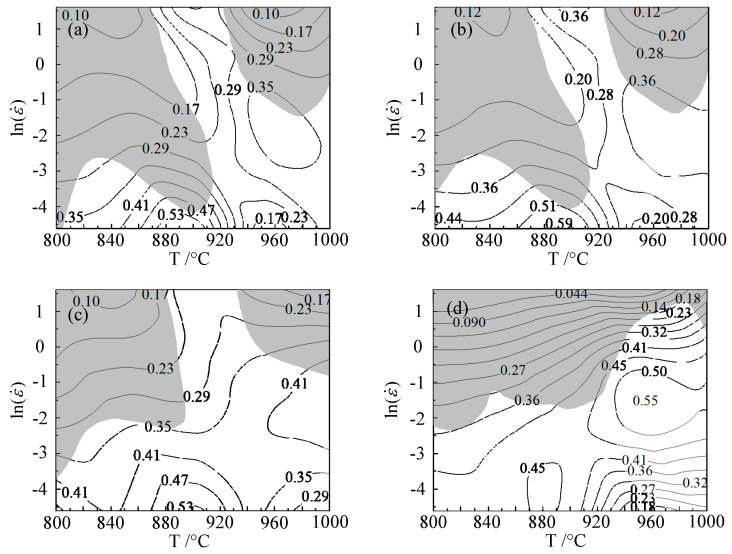
Hot processing maps at various strains: (**a**) 0.1; (**b**) 0.2; (**c**) 0.4; (**d**) 0.6.

Figure 9 shows the hot processing maps of Ti6Al4V under various strains. When the strain was 0.1, the deformation instability domains of Ti6Al4V were in either the temperature range of 800–920 °C and the strain rate range of 0.018–5 s^−1^, or the temperature range of 930–1000 °C and strain rate range of 0.22–5 s^−1^. The workability domains of the alloy were in temperature range of 860–915 °C and the strain rate range of 0.01–0.014 s^−1^, with a peak power dissipation efficiency of 0.56, or in the temperature range of 930–1000 °C and the strain rate range of 0.05–0.59 s^−1^, with a peak power dissipation efficiency of 0.52.

When the strain was 0.2, the deformation instability domains of Ti6Al4V were in the temperature range of 800–915 °C and the strain rate range of 0.03–5 s^−1^, or in the temperature range of 930–1000 °C and the strain rate range of 0.3–5 s^−1^. The workability domains of the alloy were in the temperature range of 860–915 °C and the strain rate range of 0.01–0.014 s^−1^, with a peak power dissipation efficiency of 0.59, or in the temperature range of 930–1000 °C and the strain rate range of 0.05–0.59 s^−1^, with a peak power dissipation efficiency of 0.52.

When the strain was 0.4, the deformation instability domains of Ti6Al4V were in the temperature range of 800–900 °C and the strain rate range of 0.046–5 s^−1^, or in the temperature range of 940–1000 °C and the strain rate range of 0.45–5 s^−1^. The workability domains of the alloy were in the temperature range of 840–940 °C and the strain rate range of 0.01–0.049 s^−1^, with a peak power dissipation efficiency of 0.59, or in the temperature range of 950–1000 °C and the strain rate range of 0.049–0.61 s^−1^, with a peak power dissipation efficiency of 0.53.

When the strain was 0.6, the deformation instability domains of Ti6Al4V were in the temperature range of 800–935 °C and the strain rate range of 0.13–5 s^−1^, or in the temperature range of 940–1000 °C and the strain rate range of 1.35–5 s^−1^. The workability domains of the alloy were in the temperature range of 840–920 °C and the strain rate range of 0.01–0.049 s^−1^, with a peak power dissipation efficiency of 0.59, or in the temperature range of 940–980 °C and the strain rate range of 0.11–1.65 s^−1^, with a peak power dissipation efficiency of 0.55.

It can be seen that the instability of Ti6Al4V mainly occurred in the low-temperature region and in the high-temperature and high-strain region, showing poor formability, and thus, these regions should be avoided during plastic processing. Beyond the instability domain was the safety domain. There were two workability domains of medium strain rate at high temperature and low strain rate at medium temperature in the safety domain. When the strain was 0.1, there was a lot of overlap between the workability domains and the unstable domains, which was not conducive to plastic processing. As the strain increased, the overlap became smaller. Under the condition of the strain of 0.6, the peak power dissipation region did not coincide or make contact with the instability region, and the peak power dissipation was the largest, with good formability.

**Figure 10:**
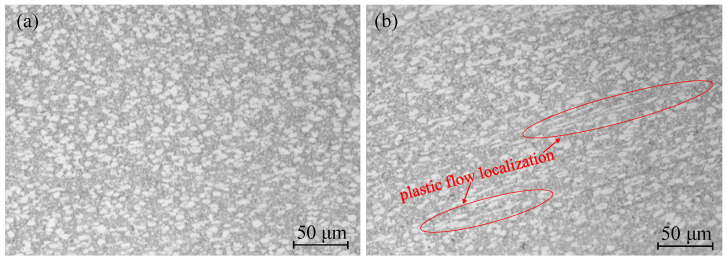
Microstructure of the safe domain and the unstable domain of Ti6Al4V: (**a**) 850 °C/0.01 s^−1^; (**b**) 900 °C/5 s^−1^.

An optical microscope was used to observe the metallographic structure of the safe domain and the unstable domain Ti6Al4V, as shown in Figure 10. As shown in Figure 10a, under the deformation condition of 850 °C/0.01 s^−1^, the Ti6Al4V titanium alloy was in a state of coexistence of the α and β phases, and dynamic recrystallization could occur in both the α and β phases. Due to the hexagonal close-packed structure of the α phase, the stacking fault energy was low, and dynamic recrystallization was more likely to occur, so it could be clearly seen that the equiaxed α grains were small, the β phase had a body-centered cubic structure, the stacking fault energy was high, and dynamic recrystallization occurred. The dislocation energy required for crystallization was high. Under the same conditions, dynamic recrystallization of the β phase could not easily occur. Therefore, it can be seen in the aforementioned figure that the dynamic recrystallization of β phase was not obvious. At 900 °C/5 s^−1^, the phenomenon of local plastic flow could be clearly observed, as shown in Figure 10b. The α phase had a long strip distribution along the flow direction, and the unstable deformation structure appeared. The safe domain microstructure and unstable microstructure of the alloy indicated that the processing properties of the alloy could be well described by the processing map. When the strain was 0.6, the optimal processing domain range comprised temperatures of 840–920 °C and strain rates of 0.01–0.049 s^−1^. The second most suitable processing domain range involved temperatures of 940–980 °C and strain rates of 0.11–1.65 s^−1^.

## 4. Conclusions

In this paper, high-temperature compression tests of Ti6Al4V alloys on Gleeble-1500D at temperatures of 800–1000 °C and strain rates of 0.01–5 s^−1^ were carried out, and the flow curves were obtained at high temperatures. When the temperature was below 850 °C, the microstructure of Ti6Al4V was dominated by the α phase, a hexagonal close-packed structure with low stacking fault energy and easy dynamic recrystallization. However, with the increasing of the temperature (above 850 °C), more and more α-phase transformed into β-phase (a body-centered cubic structure), and the β-phase’s stacking fault energy was high, which made the recrystallization difficult. Therefore, when the deformation temperature was above 950 °C, the β phase was dominant in the Ti6Al4V alloy, and the flow stress showed the characteristics of dynamic recovery.

Based on the inverse optimization method, a new parameter determination method for the constitutive model is proposed. The high-temperature Hansel-Spittel model parameters of Ti6Al4V alloy were identified by means of response surface methodology and the genetic optimization algorithm. The values of the correlation coefficient (*R*) and the average absolute relative error (*AARE*) were 0.9999 and 0.0484, respectively, which indicated that the stress calculated by the Hansel-Spittel model was in very good agreement with the experimental results.

In order to research the formability of Ti6Al4V alloy, the dynamic material model was used to establish the thermal processing map of the material under strain levels of 0.1–0.6. The results show that when the strain was 0.6, the power dissipation values of the two formable regions were 0.59 and 0.55, respectively, and thus, were higher than those of the processing diagram for strain levels below 0.6. Furthermore, the optimal hot processing area was the first domain (840–920 °C/0.01–0.049 s^−1^) and the second domain (940–980 °C/0.11–1.65 s^−1^). The microstructural photos of Ti6Al4V were observed with an optical microscope. At 850 °C/0.01 s^−1^, α phase with fine grains and β phase with good plasticity were obtained; these had good formability and verified the accuracy of the hot working map.

## Figures and Tables

**Table 1:** Chemical composition of Ti6Al4V (wt, %).

Ti	Al	V	Fe	C	N	H	O
Bal.	6.11	3.93	0.131	0.016	<0.005	<0.001	0.113

**Table 2:** Hansel-Spittel model orthogonal test factor levels.

Level	Factor								
	A	m_1_	m_2_	m_3_	m_4_	m_5_	m_7_	m_8_	m_9_
1	345	−0.0092	−0.01	−0.19	−0.0032	0.002	−2.3	0.00039	0.9
2	350	−0.0093	−0.03	−0.2	−0.0033	0.0025	−2.4	0.0004	1
3	355	−0.0094	−0.05	−0.21	−0.0034	0.003	−2.5	0.00041	1.1
4	360	−0.0095	−0.07	−0.22	−0.0035	0.0035	−2.6	0.00042	1.2

**Table 3:** Design arrangement and response of orthogonal experiment.

Group Number	Parameter									Result
	A	m_1_	m_2_	m_3_	m_4_	m_5_	m_7_	m_8_	m_9_	O(f)
1	345	−0.0092	−0.01	−0.19	−0.0032	0.002	−2.3	0.00039	0.9	0.624348
2	345	−0.0093	−0.03	−0.2	−0.0033	0.0025	−2.4	0.0004	1	0.337491
3	345	−0.0094	−0.05	−0.21	−0.0034	0.003	−2.5	0.00041	1.1	0.03274
4	345	−0.0095	−0.07	−0.22	−0.0035	0.0035	−2.6	0.00042	1.2	0.511145
5	350	−0.0092	−0.01	−0.2	−0.0033	0.003	−2.5	0.00042	1.2	0.815538
6	350	−0.0093	−0.03	−0.19	−0.0032	0.0035	−2.6	0.00041	1.1	0.008196
7	350	−0.0094	−0.05	−0.22	−0.0035	0.002	−2.3	0.0004	1	0.39574
8	350	−0.0095	−0.07	−0.21	−0.0034	0.0025	−2.4	0.00039	0.9	0.648502
9	355	−0.0092	−0.03	−0.21	−0.0035	0.002	−2.4	0.00041	1.2	0.474217
10	355	−0.0093	−0.01	−0.22	−0.0034	0.0025	−2.3	0.00042	1.1	0.029997
11	355	−0.0094	−0.07	−0.19	−0.0033	0.003	−2.6	0.00039	1	0.314332
12	355	−0.0095	−0.05	−0.2	−0.0032	0.0035	−2.5	0.0004	0.9	0.58867
13	360	−0.0092	−0.03	−0.22	−0.0034	0.003	−2.6	0.0004	0.9	0.554467
14	360	−0.0093	−0.01	−0.21	−0.0035	0.0035	−2.5	0.00039	1	0.234209
15	360	−0.0094	−0.07	−0.2	−0.0032	0.002	−2.4	0.00042	1.1	0.083837
16	360	−0.0095	−0.05	−0.19	−0.0033	0.0025	−2.3	0.00041	1.2	0.305932
17	345	−0.0092	−0.07	−0.19	−0.0035	0.0025	−2.5	0.0004	1.1	0.02964
18	345	−0.0093	−0.05	−0.2	−0.0034	0.002	−2.6	0.00039	1.2	0.269416
19	345	−0.0094	−0.03	−0.21	−0.0033	0.0035	−2.3	0.00042	0.9	0.55
20	345	−0.0095	−0.01	−0.22	−0.0032	0.003	−2.4	0.00041	1	0.376197
21	350	−0.0092	−0.07	−0.2	−0.0034	0.0035	−2.3	0.00041	1	0.136266
22	350	−0.0093	−0.05	−0.19	−0.0035	0.003	−2.4	0.00042	0.9	0.562345
23	350	−0.0094	−0.03	−0.22	−0.0032	0.0025	−2.5	0.00039	1.2	0.27384
24	350	−0.0095	−0.01	−0.21	−0.0033	0.002	−2.6	0.0004	1.1	0.187258
25	355	−0.0092	−0.05	−0.21	−0.0032	0.0025	−2.6	0.00042	1	0.300622
26	355	−0.0093	−0.07	−0.22	−0.0033	0.002	−2.5	0.00041	0.9	0.634336
27	355	−0.0094	−0.01	−0.19	−0.0034	0.0035	−2.4	0.0004	1.2	0.786602
28	355	−0.0095	−0.03	−0.2	−0.0035	0.003	−2.3	0.00039	1.1	0.035255
29	360	−0.0092	−0.05	−0.22	−0.0033	0.0035	−2.4	0.00039	1.1	0.061428
30	360	−0.0093	−0.07	−0.21	−0.0032	0.003	−2.3	0.0004	1.2	1.17284
31	360	−0.0094	−0.01	−0.2	−0.0035	0.0025	−2.6	0.00041	0.9	0.659364
32	360	−0.0095	−0.03	−0.19	−0.0034	0.002	−2.5	0.00042	1	0.460461

**Table 4:** Analysis of variance of the response surface model.

	DOF	SS	MS	F	*p*
model	27	2.34076	0.086695	6.16	0.044 *
A	1	0.01336	0.013362	0.95	0.385
m_1_	1	0.16988	0.169881	12.08	0.025 *
m_2_	1	0.233	0.233004	16.56	0.015 *
m_3_	1	0.08033	0.080328	5.71	0.075
m_4_	1	0.18458	0.184576	13.12	0.022 *
m_5_	1	0.13818	0.138182	9.82	0.035 *
m_7_	1	0.00505	0.005048	0.36	0.581
m_8_	1	0.01566	0.015663	1.11	0.351
m_9_	1	0.39514	0.395143	28.09	0.006 **
$m_{2}^{2}$	1	0.03172	0.031718	2.25	0.208
$m_{3}^{2}$	1	0.05035	0.050352	3.58	0.131
$m_{4}^{2}$	1	0.20433	0.204329	14.52	0.019 *
$m_{5}^{2}$	1	0.00602	0.00602	0.43	0.549
$m_{7}^{2}$	1	0.00363	0.00363	0.26	0.638
$m_{8}^{2}$	1	0.21908	0.219076	15.57	0.017 *
$m_{9}^{2}$	1	0.38767	0.38767	27.56	0.006 **
Am_1_	1	0.02075	0.020747	1.47	0.291
Am_2_	1	0.05836	0.05836	4.15	0.111
Am_3_	1	0.28912	0.289116	20.55	0.011 *
Am_4_	1	0.0653	0.065299	4.64	0.097
Am_5_	1	0.15959	0.159592	11.34	0.028 *
Am_8_	1	0.02056	0.020563	1.46	0.293
m_1_m_2_	1	0.19687	0.196867	13.99	0.02 *
m_1_m_3_	1	0.20296	0.20296	14.43	0.019 *
m_1_m_5_	1	0.20578	0.205777	14.63	0.019 *
$m_{2}^{3}$	1	0.22876	0.228762	16.26	0.016 *
$m_{3}^{3}$	1	0.05061	0.050614	3.6	0.131
error	4	0.05627	0.014068		
total	31	2.39703			
*R*^2^ = 0.9952					

* means significant (0.01 < *p* < 0.05), ** means extremely significant (*p* < 0.01).

**Table 5:** Optimal parameters of the Ti6Al4V constitutive model.

Parameter	A	m_1_	m_2_	m_3_	m_4_	m_5_	m_7_	m_8_	m_9_
optimal value	356.307	−0.00935	−0.032	−0.2007	−0.0033	0.00317	−2.356	0.00041	1.089

## Data Availability

Not applicable.
